# Development of the "GeneSYS" database system to support trial data capture and conduct

**DOI:** 10.1186/1745-6215-14-S1-P66

**Published:** 2013-11-29

**Authors:** D Hutton, N Smith, H Cappel-Porter, C Saw, CA Rogers

**Affiliations:** 1 University of Bristol, Bristol, UK

## Background

Data from the recent UK-wide registration process for clinical trials units (CTU) revealed that a significant proportion of units use in-house developed solutions despite the availability of numerous commercial and open source solutions, suggesting these solutions may not meet individual trial requirements. Recognising the need to develop flexible database solutions quickly, and that are easily maintained throughout the life cycle of a study, the CTU database development team conceived and developed the "GeneSYS" database solution.

## Methods

GeneSYS allows clinical trial databases to be created without any coding via an intuitive web interface. It simplifies the creation of electronic CRFs, even with complex data validation rules. The web viewer provides an easy to use interface for data collection.

## Results

First released in Nov 2012, GeneSYS currently supports eight trials, including two multi-centre trials and two trials run externally to the CTU. It is hosted on a secure NHS server and accessed via the NHS clinical portal. Initial feedback has been very positive. Trial database development time has reduced significantly, thereby increasing our capacity to support more trials. We are investigating developing a mobile viewer so that electronic capture could become the primary data source.

## Conclusion

GeneSYS has been a success; it has the potential to support a wider range of trials and to make database creation more efficient and intuitive enough for non-technical staff to author.

